# Green tea extract decreases starch digestion and absorption from a test meal in humans: a randomized, placebo-controlled crossover study

**DOI:** 10.1038/srep12015

**Published:** 2015-07-30

**Authors:** Klaudia Lochocka, Joanna Bajerska, Aleksandra Glapa, Ewa Fidler-Witon, Jan K. Nowak, Tomasz Szczapa, Philip Grebowiec, Aleksandra Lisowska, Jaroslaw Walkowiak

**Affiliations:** 1 Department of Pediatric Gastroenterology and Metabolic Diseases, Poznan University of Medical Sciences, Poznan, Poland; 2 Department of Human Nutrition and Hygiene, Poznan University of Life Sciences, Poznan, Poland; 3 Department of Neonatology, Poznan University of Medical Sciences, Poznan, Poland

## Abstract

Green tea is known worldwide for its beneficial effects on human health. However, objective data evaluating this influence in humans is scarce. The aim of the study was to assess the impact of green tea extract (GTE) on starch digestion and absorption. The study comprised of 28 healthy volunteers, aged 19 to 28 years. In all subjects, a starch ^13^C breath test was performed twice. Subjects randomly ingested naturally ^13^C-abundant cornflakes during the GTE test (GTE 4 g) or placebo test. The cumulative percentage dose recovery (CPDR) was significantly lower for the GTE test than for the placebo test (median [interquartile range]: 11.4% [5.5–15.5] vs. 16.1% [12.7–19.5]; p = 0.003). Likewise, CPDR expressed per hour was considerably lower in each point of the measurement. In conclusion, a single dose of green tea extract taken with a test meal decreases starch digestion and absorption.

Obesity and its associated comorbidities remain a global health problem. A study by Ng *et al*. estimated that 2.1 billion adults were overweight in 2013^1^. The main reasons for this are the imbalance between dietary intake and energy expenditure, sedentary lifestyle, and improper dietary habits. Additionally, high intake of simple carbohydrates may contribute to weight gain and increased postprandial glycemia^2^,^3^. One seemingly crucial and viable strategy towards the reduction or normalization of body weight would be to reduce the intake of simple carbohydrates.

There are many nutritional and, to a lesser extent, pharmacological strategies that result in successful body fat mass reduction. Epidemiological evidence and several randomized controlled intervention trials have shown that habitual tea consumption (especially green tea) has a positive effect on health^4^,^5^,^6^. Green tea is known to exert anti-obesity activity like reduction of adipocyte differentiation and proliferation, lipogenesis, fat mass, body weight and fat absorption^6^.

Green tea is brewed from the unfermented dried leaves of the plant *Camellia sinensis*, which contains a wide range of phytochemicals. The putative anti-obesity effects have been most commonly attributed to the polyphenolic fraction of green tea, especially the catechins. These include: epigallocatechin gallate (EGCG), epicatechin gallate (ECG), epigallocatechin (EGC), epicatechin (EC), and catechin (C). EGCG, which represents 50–80% of the total catechin content, is the most abundant catechin in green tea^7^,^8^. Scientific evidence indicates that green tea extracts, its polyphenols, including EGCG, lower the level of blood glucose and thus have anti-diabetic effects^9^,^10^,^11^,^12^. The supposed mechanism of action is the inhibition of α-amylase and α-glucosidase, which are responsible for starch hydrolysis^13^. Moreover, EGCG is associated not only with anti-obesity activity, but also anti-bacterial, anti-viral, and anti-cancer effects^5^,^14^.

The effect of green tea extract (GTE) on starch digestion and absorption is widely discussed. Nevertheless, data evaluating this influence in humans are scarce. Isotope breath tests prove to be useful as a non-invasive method assessing breakdown of carbohydrates^15^. The aim of this study was to assess the impact of GTE on starch digestion and absorption with the use of a breath test in humans.

## Results

In most subjects (78.6%), the decreased starch digestion and absorption due to GTE was rapid and the aforesaid effect persisted until the last measure. Cumulative percentage dose recoveries (CPDRs) were significantly lower for the GTE test than for the placebo test (median [quartile distribution]: 11.4% [5.5–15.5] vs. 16.1% [12.7–19.5]; p = 0.003) (Table 1). Likewise, the CPDR expressed per hour was considerably lower in each point of the measurement (Table 2).

## Discussion

The obtained data show that a single dose of GTE ingested with the test meal may limit the digestion and absorption of dietary starch in humans. The dose of GTE (4 g, EGCG content −257.6 mg), established according to pharmacokinetic studies, is equivalent to at least several cups of green tea^16^,^17^. As the usual daily intake of green tea is smaller than the dose used in our study, its effect may be not as pronounced. Some commercially available green teas may differ in total amount of polyphenols, even to as little as 15% polyphenols. To the best of our knowledge, this is the first study evaluating the effect of pure GTE on the digestion and absorption of starch with the use of a plausible method.

To study the effect of physicochemical characteristics of starch digestion, we used a CO_2_ starch breath test. Lacroix *et al*. used this method for the first time in measuring the ^13^CO_2_ excretion in breath after ingestion of naturally ^13^C-labelled glucose^18^. The principle of the method is based on the fact that the glucose, previously hydrolyzed from starch, is absorbed and further metabolized to CO_2_. Breath tests are considered to be a reliable method to study the carbohydrate absorption, and are widely used in gastrointestinal studies^19^.

The key enzyme in the digestive system –α-amylase (of pancreatic as well as salivary origin) breaks down the starch contained in cornflakes. In the initial step, α-amylase catalyzes the hydrolysis of starch to smaller oligosaccharides consisting of maltose, maltotriose, and a number of α-(1–6)- and α-(1–4)-oligoglucans, which are further degraded by α-glucosidases to glucose. This process may lead to the elevated post-prandial hyperglycemia occurring in diabetes. Hence, effective inhibition of enzymes such as α-amylase is crucial in the control of this disease. Most inhibitors, such as acarbose, cause serious side effects (diarrhea, vomiting). For this reason, much attention has been paid to herbs and plant extracts that offer similar benefits without the side effects^20^,^21^. Although high doses of EGCG (e.g. 800 mg) might cause mild headache and fatigue^17^, the usage of 257.6 mg EGCG did not cause any side effects in the subjects during and after the study beyond leaving a taste of bitterness in 5 subjects.

Honda and Hara have previously reported that GTE inhibited human salivary α-amylase^22^. Another study evaluated the inhibitory effects of GTE and its catechins on α-amylase and α-glucosidase activity in comparison with acarbose. GTE (especially EGCG) was a stronger inhibitor than the latter^20^. Similar results evaluating EGCG activity were obtained in starch-fed mice^23^. Despite a few studies conducted in animal models^24^,^25^,^26^,^27^, little is known about the impact of pure GTE on starch digestion and absorption in humans. The research carried out in healthy Asians provided evidence that a beverage containing 0.1 g black, 0.1 g green and 1.0 g mulberry teas caused carbohydrate malabsorption of 25% (~60 kcal) compared to placebo in healthy adults as assessed by breath hydrogen analysis^28^. Although suggestive of an influence of green tea on inhibiting starch digestion and absorption, this data is confounded by the complicated mixture of three teas and the use of a rice-based meal rather than pure starch. In contrast to the aforementioned study we used GTE alone. We avoided different biological interactions between compounds of three teas this way. The variety of green tea extract components as well as their effects on humans have been extensively studied. The study of Gao *et al*. shows that GTE and its polyphenols namely EGCG, strongly suppress the α-glucosidase *in vitro*^29^. Based on the half maximal inhibitory concentration (IC_50_) values, GTE, green tea polyphenols and EGCG alone demonstrate 800–1000 times the efficacy of acarbose (IC_50_ values of GTE, green tea polyphenols and EGCG against α-glucosidase were 4.421 ± 0.018, 10.019 ± 0.017 and 5.272 ± 0.009 respectively, whereas of acarbose against α-glucosidase values were 4,822.783 ± 26.042). In regard to α-amylase, it was not strongly inhibited by these substances. Similar results were obtained in the study of Yang *et al*. who proved, consistently with the previous report, that the inhibition of α-glucosidase by tea polyphenols is noncompetitive^13^,^30^. Furthermore, Gao *et al*. also reported that the combination of acarbose and GTE, EGCG, or green tea polyphenols show combined inhibitory effects at certain concentrations^29^. Therefore, it could be expected that joined therapy with GTE or green tea polyphenols or EGCG may diminish the dose of acarbose needed in therapy, hence weaken the side effects of acarbose alone. The aforesaid findings indicate that green tea or functional food based on green tea could be applicable for complementary therapy in postprandial hyperglycemia.

We used naturally ^13^C abundant cornflakes as a source of starch. The physical form of starch, the method of its processing or the size of the particle are a vital factors that may influence the hydrolysis and glycemic response in subjects^31^. Commercially available cereals used in the study, were produced via an extrusion process, which gives starch a high degree of gelatinization. Based on the study of Hiele *et al*. starch in this formation is more rapidly hydrolysed than native starch^19^. Maize, a representative of C4 photosynthesizing plants incorporates more ^13^C atoms into the starch than C3 plants do (e.g. European grain, potatoes, rice). The typical Polish diet (in contrast to the American diet) contains foodstuffs like potatoes, rye, wheat, beet sugar, and (to lesser extent) maize, therefore maintaining the conditions of the test was easier.^15^

Our data suggest that the use of GTE is a viable alternative to pharmaceutical inhibitors of glucoside hydrolase enzymes. This plant extract is widely available, inexpensive, and well tolerated, so it has potential utility for weight control and the treatment of diabetes. Our study supports the concept that pure GTE inhibits starch digestion and absorption. However, the clinical significance of each green tea catechin and the exact mechanism responsible for this action in humans remain to be determined.

## Methods

### Subjects

The study comprised of 28 healthy, adult volunteers (19 women and 9 men, aged 19–26 years old) recruited from Vocational Technical High School for Computer Science in Nakło nad Notecią, Poland (Table 3). All subjects were recruited after they presented at an appointed meeting and showed willingness to participate the study, which was thereafter confirmed by informed written consent. The health status was defined as such: no physical complaints in the month preceding the study, no acute or chronic disease, no current pharmacotherapy, no past hospitalizations for gastroenterological indications, and good nutritional status defined as weight, height, and BMI within normal reference values. As the subjects were to ingest milk, a hydrogen-methane breath test was performed in each subject, to exclude lactose malabsorption. In the first week of study, some of the participants ingested the test meal with GTE while the others ingested placebo. The order of the first test (GTE/placebo test) was determined according to the randomization list. The second test followed in a crossover manner. One week later, subjects who took GTE in the previous test were given placebo, and those receiving placebo in the previous week were given GTE. In this way subjects were internal self-controls to themselves.

Exclusion criteria comprised: celiac disease, exocrine pancreatic insufficiency^32^,^33^ and other gastrointestinal diseases, pharmacotherapeutics that might affect digestion and absorption of carbohydrates, antibiotic therapy within the preceding month, and the use of beverages composed of green tea within the preceding month.

### Protocol

All subjects fasted for 12-hours. After collection of baseline breath samples for ^13^CO_2_ analysis, the subjects ingested a test meal consisting naturally ^13^C-abundant cornflakes (50 g cornflakes + 100 ml low- fat milk). The total energy load of the test meal amounted to 234 kcal, and it consisted of 7.0 g protein, 46.5 g carbohydrates, and 2.2 g fat. Concurrently with the test meal, a group of the subjects ingested GTE (GTE 4 g, EGCG content –257.6 mg) in a powder form enclosed in digestible starch wafer, whereas the others ingested an empty starch wafer (placebo control). The subjects were assigned to the groups randomly. Breath samples were collected at 30, 60, 90, 120, 150, 180, 210, and 240 minutes after the test meal ingestion. One week later, the procedure was repeated, though the subjects ingested the opposite preparation from that ingested in the initial study.

The subjects were also instructed to not eat any food with a naturally increased ^13^C content, such as products made of maize, cane sugar, pineapple, kiwi fruit for 5 days preceding the examination. The participants were asked not to consume any additional food or beverages and not to perform any physical activity in order to limit the glucose oxidation level and to only obtain the rate of starch hydrolysis. The isotope ratio ^12^CO_2_/^13^CO_2_ of breath samples was measured with the use of the IRIS isotope apparatus (Wagner Analysen Technik GmbH, Bremen, Germany). CPDR was considered to reflect digestion and absorption of dietary starch.

GTE was prepared according to the protocol described by Bajerska *et al*.^34^ The tea leaves (100 g) were ground and subsequently boiled in double distilled water (1000 mL), then stirred for 15 minutes at 70 ^o^C (repeated 3 times). Collected extracts were filtered, centrifuged for 15 minutes, frozen and lyophilized under a vacuum. (Elena, Zelazkow, Poland). One gram of green tea aqueous extract dry matter contained 7.0% EGCG, 4.1% EGC, and 1.8% ECG, as determined by high-performance liquid chromatography (HPLC).

The study was carried out in accordance with the Declaration of Helsinki. Every subject provided written consent to participate after being informed about the aim and protocol of the research. The study design was approved by the Bioethical Committee of Poznan University of Medical Sciences, Poland (protocols 605/12 and 752/13).

### Statistical analysis

Results are expressed as medians and interquartile ranges. The statistical significance of differences between GTE and placebo tests was determined with the use of the Wilcoxon rank-sum test. A level of significance was set at *p* < 0.05. Statistical analysis was performed using STATISTICA 10.0 (StatSoft Inc., Tulsa, USA).

## Additional Information

**How to cite this article**: Lochocka, K. *et al*. Green tea extract decreases starch digestion and absorption from a test meal in humans: a randomized, placebo-controlled crossover study. *Sci. Rep*. **5**, 12015; doi: 10.1038/srep12015 (2015).

## Figures and Tables

**Table 1:** Starch digestion and absorption based on cumulative percentage ^13^C dose recovery (CDPR).

CPDR [%^13^C]								
Minutes	30	60	90	120	150	180	210	240
GTE	0	0.1	1.0	2.7	5.1	7.9	10.0	11.4
	[0–0.3]	[0–0.7]	[0.4–2.2]	[1.6–4.5]	[2.8–7.3]	[4.0–10.2]	[4.4–12.8]	[5.5–15.5]
Placebo	0.1	0.7	2.3	4.7	7.8	11.0	13.8	16.1
	[0–0.3]	[0.1–1.3]	[1.4–3.4]	[3.7–6.0]	[6.6–9.2]	[9.2–12.8]	[11.0–16.3]	[12.7–19.5]
Statistical significance (p)	0.027	0.015	0.018	0.014	0.006	0.003	0.003	0.003

Median values and interquartile ranges are presented.

*GTE – green tea extract*.

**Table 2:** Starch digestion and absorption based on ^13^C dose recovery expressed per hour.

Dose recovery per hour [%^13^C]								
Minutes	30	60	90	120	150	180	210	240
GTE	0	1.0	3.2	4.1	4.8	4.6	3.7	3.6
	[0–0.4]	[0.5–1.8]	[2.0–4.5]	[2.9–4.8]	[2.6–6.8]	[2.3–5.7]	[2.7–5.6]	[1.8–5.1]
Placebo	0.5	2.2	4.3	5.5	6.1	6.2	5.7	4.6
	[0–1.1]	[1.0–3.5]	[3.5–4.9]	[4.8–6.8]	[5.2–7.8]	[5.1–7.2]	[3.3–6.9]	[2.6–6.5]
Statistical significance (p)	0.017	0.030	0.042	0.002	0.005	0.006	0.001	0.020

Median values and interquartile ranges are presented.

*GTE – green tea extract*.

**Table 3:** Group characteristics (n = 28).

Parameter	Median	Interquartile range
Age [years]	19.0	19.0–19.7
Body weight [kg]	60.7	55.0–69.2
Height [cm]	170	165–177
BMI [kg/m^2^]	20.3	19.1–23.3

*BMI – Body Mass Index*.
